# Mapping oxygen concentration in the awake mouse brain

**DOI:** 10.7554/eLife.12024

**Published:** 2016-02-02

**Authors:** Declan G Lyons, Alexandre Parpaleix, Morgane Roche, Serge Charpak

**Affiliations:** 1 Institut National de la Santé et de la Recherche Médicale, U1128, Paris, France; 2 Laboratory of Neurophysiology and New Microscopies, Université Paris Descartes, Paris, France; University of California, San Diego, United States

**Keywords:** two-photon microscopy, oxygen, phosphorescence, olfactory bulb, cortex, blood flow, Mouse

## Abstract

Although critical for brain function, the physiological values of cerebral oxygen concentration have remained elusive because high-resolution measurements have only been performed during anesthesia, which affects two major parameters modulating tissue oxygenation: neuronal activity and blood flow. Using measurements of capillary erythrocyte-associated transients, fluctuations of oxygen partial pressure (Po_2_) associated with individual erythrocytes, to infer Po_2_ in the nearby neuropil, we report the first non-invasive micron-scale mapping of cerebral Po_2_ in awake, resting mice. Interstitial Po_2_ has similar values in the olfactory bulb glomerular layer and the somatosensory cortex, whereas there are large capillary hematocrit and erythrocyte flux differences. Awake tissue Po_2_ is about half that under isoflurane anesthesia, and within the cortex, vascular and interstitial Po_2_ values display layer-specific differences which dramatically contrast with those recorded under anesthesia. Our findings emphasize the importance of measuring energy parameters non-invasively in physiological conditions to precisely quantify and model brain metabolism.

**DOI:** http://dx.doi.org/10.7554/eLife.12024.001

## eLife digest

Brain cells need a constant supply of oxygen to fuel their activities. This oxygen is delivered by the flow of blood through the vessels in the brain. If the blood flow to brain tissue is cut off as happens in stroke, or if an individual stops breathing, the brain becomes deprived of oxygen and brain cells will be damaged and die. To better understand how the brain works in health and disease, scientists need to learn how much oxygen the blood must deliver to the brain tissue to adequately support the activities of brain cells.

Many studies have measured oxygen levels in the brain. However, these studies have looked only roughly and taken measurements from large areas of the brain, or they have involved animals receiving anesthesia, which can alter blood flow and oxygen use in the brain. Recently, scientists discovered that they could measure oxygen concentration at high detail in the brain of anesthetized rodents with a specialized microscope, by using molecules that emit light at a rate that depends on the local oxygen concentration.

Now, Lyons et al. have shown that this same technique can be used in mice that are awake. First, a piece of the skull was replaced with glass to create a small transparent window. Then, the animals were allowed to recover for a few weeks, and were trained to get them used to how they would be handled during the experiments. After this period, the oxygen concentrations and blood flow in different parts of the mouse brains were measured in fine detail using the microscope while the animals were awake and relaxed.

The experiments showed that oxygen levels in awake resting mice are actually lower than in anesthetized mice, and that oxygen levels differ between different parts of the mouse brain. This first detailed look at oxygen levels in the brain of awake animals will likely lead to more studies. For example, future studies may look at how quickly the brain uses oxygen under normal conditions and what happens in the brain during disease.

**DOI:** http://dx.doi.org/10.7554/eLife.12024.002

## Introduction

To understand the relationship between brain oxygenation and diseases associated with hypoxia or ischemia, it is important to first determine what fixes the resting value of tissue Po_2_, that is, the concentration of oxygen in the interstitium that bridges oxygen delivery from erythrocytes to oxygen consumption by mitochondria. Numerous methods have been used to monitor brain oxygenation, and the most spatially resolved approaches have long relied on fine Clark-type electrodes (for review see Ndubuizu and LaManna, 2007), which have been used to report resting-state Po_2_ and local oxygen consumption in various brain regions (Lecoq et al., 2009; Masamoto et al., 2003; Offenhauser et al., 2005; Thompson et al., 2003) during neuronal activation. However, these electrodes are invasive, do not faithfully report Po_2_ in vessels and cannot easily be used to determine Po_2_ in physiological conditions, that is, in awake, unstressed animals, avoiding the use of anesthetics. As anesthetics affect resting and evoked neuronal and astrocyte activity, arterial blood pressure and cerebral blood flow, the physiological values of cerebral interstitial Po_2 _and their relationship to blood flow parameters in capillaries remain unknown.

Recently, a two-photon phosphorescent probe PtP-C343 has been generated (Finikova et al., 2007, 2008) and two-photon phosphorescence lifetime microscopy (2PLM) has been used to obtain depth-resolved, micron-scale measurements of Po_2_ in the anesthetized rodent brain (Devor et al., 2011; Lecoq et al., 2011; Parpaleix et al., 2013; Sakadzić et al., 2010; Sakadžić et al., 2014). In addition, by detecting single red blood cells (RBCs) during Po_2_ measurement, we demonstrated the possibility of simultaneously monitoring blood flow and Po_2_ in capillaries (Lecoq et al., 2011) and of detecting erythrocyte-associated transients (EATs), Po_2_ fluctuations associated with each individual erythrocyte flowing in capillaries, which were first reported in mesentery capillaries (Golub and Pittman, 2005). We showed that in olfactory bulb glomeruli of anesthetized mice, one parameter of EATs, the Po_2_ in between two red blood cells (Po_2_InterRBC), is at equilibrium with, and thus reports, the Po_2_ in the nearby neuropil (Parpaleix et al., 2013). This result implied that measurements of Po_2_InterRBC could provide a powerful tool to non-invasively map local interstitial oxygen concentration in the brain of awake animals.

Here, we report that in both the olfactory bulb glomerular layer and the somatosensory cortex of unstressed, awake, resting mice, the interstitial Po_2_ (equivalent to Po_2_InterRBC) has the same mean value of ~23 mm Hg, spanning over a range of about 40 mm Hg. This contrasts with the large differences of capillary hematocrit and RBC flow values observed between the two brain regions. In addition, we find that in the cortex capillary and interstitial Po_2_ values display layer-specific differences, being lower in layer I than in layer II/III or layer IV. We also find that hemoglobin in brain capillaries is highly saturated with oxygen. Finally, we show that in both brain regions, the interstitial Po_2_ is much lower during wakefulness than under isoflurane anesthesia.

## Results

### Habituation of mice to head-restraint

To ensure that we measured Po_2_ in real physiological conditions, that is, in awake, unstressed animals, each animal was habituated to all the conditions present during 2PLM Po_2_ measurements for several weeks prior to the experiments (see methods for detailed training procedures). In brief, over the course of 2–3 days, each mouse was habituated to handling, and trained to run on a treadmill placed in its cage. A titanium bar was then surgically attached to the cranium and then a cranial window implanted over the region of interest, either the olfactory bulb or the somatosensory cortex. Then, over 2–4 weeks, the mouse was progressively habituated to being head-fixed, via the attached bar, in the dark, below the objective of the two-photon microscope, and with the limbs and body free to move on the treadmill. Habituation was achieved when the animal remained calm for periods >1 hr in the set-up with short bouts of running (~30 s). On the day of recording, the animal was briefly anesthetized (2% isoflurane, <5 min) and the oxygen sensor PtP-C343 was injected intravenously. The animal was returned to its home cage, and after a delay of 90–120 min, Po_2_ recordings sessions of 1–3 hr commenced. Each animal underwent 1–3 recording sessions per day over the course of 2–7 days, with breaks of at least several hours between each session. Note that similar Po_2_ values were obtained from one day to the next and between sessions occurring the same day (without reinjection of PtP-C343).

### Characterization of EAT properties (Po_2_ values) in the olfactory bulb of awake resting mice

**Figure 1.**
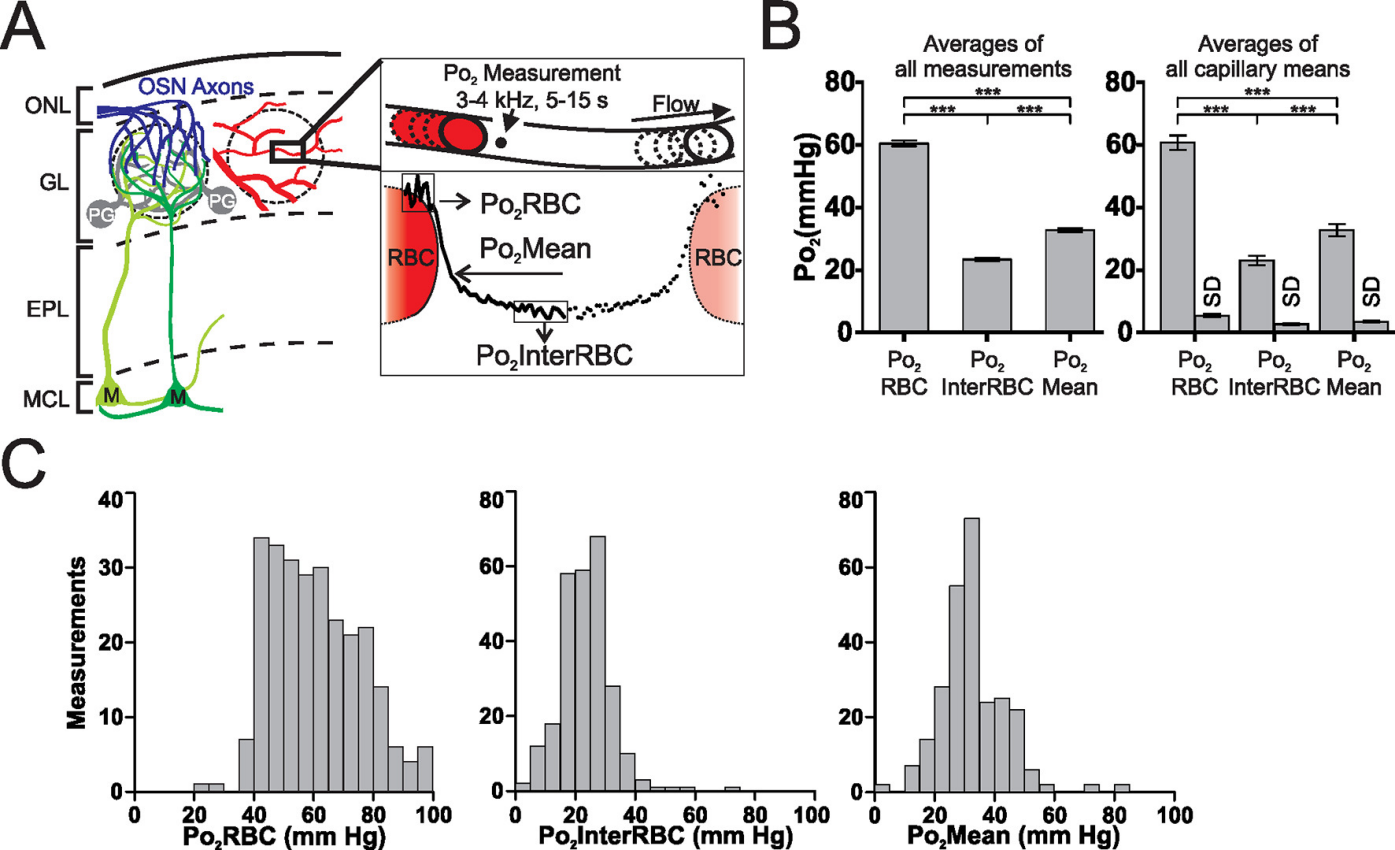
Erythrocyte-associated transients (EATs) in the olfactory bulb glomerular layer of the awake mouse. (**A**) Left panel, schematic diagram of the organisation of the olfactory bulb. OSN: olfactory sensory neuron, PG: periglomerular neuron, M: mitral cell, ONL: olfactory nerve layer, GL: glomerular layer, EPL: external plexiform layer, MCL: mitral cell layer. Right panel, top, schematic illustrating the 2PLM Po_2_ measurement procedure in capillaries. Bottom, diagram showing Po_2_ values extracted from EATs. The continuous trace represents the Po_2_ profile relative to the RBC border in one selected capillary: Po_2_ at the RBC border (Po_2_RBC, in this case 47.2 mm Hg), Po_2_ at distance from a RBC (Po_2_InterRBC, in this case 8.6 mm Hg) which gives an estimate of Po_2_ in the interstitium of the glomerular layer, and average Po_2_ in the capillary (Po_2_Mean, in this case 19 mm Hg). (**B**) Multiple (~4–8) measurements were made in each capillary. The Po_2_InterRBC is significantly lower than both Po_2_Mean and Po_2_RBC, whether calculated on all measurements (the mean value is the average of all measurements pooled from all capillaries assessed, n = 262, left panel), or on the mean values from each capillary (the mean value is the average of the single mean values for each of the capillaries, n=38, right panel). SD (the average of the SD values for each capillary, presented as mean standard deviation ± SEM) illustrates the fluctuations of Po_2_ values in each capillary across the multiple measurements. Data presented as mean ± SEM. *p<0.05, ***p<0.001, Kruskal-Wallis test with 2-tailed Dunn's multiple comparison post-hoc test. (**C**) Frequency distributions of all measurements of Po_2_RBC (left panel), Po_2_InterRBC (middle panel), and Po_2_Mean (right panel). 5 mm Hg bin width. For all plots n = 5 mice. **DOI:** http://dx.doi.org/10.7554/eLife.12024.003

Using our previous approach (Lecoq et al., 2011; Parpaleix et al., 2013), we characterized EATs in 38 capillaries (n = 5 animals) from the glomerular layer of awake resting mice (Figure 1). Po_2_ measured at the RBC border (Po_2_RBC) was significantly larger than at mid-distance between two RBCs (Po_2_InterRBC). Po_2_Mean, which was intermediate between these two values, is the average Po_2_ measured in a capillary without taking into account the existence of EATs, and is the only capillary Po_2_ value that has commonly been reported in the brain (Sakadzić et al., 2010; Vovenko, 1999). Several measurements were made in each capillary but the average values of Po_2_RBC, Po_2_InterRBC and Po_2_Mean were similar whether calculated on all measurements or on all capillaries (262 measurements: Po_2_RBC = 60.5 ± 0.9 mm Hg; Po_2_InterRBC = 23.4 ± 0.5 mm Hg; Po_2_Mean = 32.8 ± 0.7 mm Hg. 38 capillaries: Po_2_RBC = 60.6 ± 2.3 mm Hg; Po_2_InterRBC = 23 ± 1.5 mm Hg; Po_2_Mean = 32.7 ± 1.9 mm Hg) (Figure 1B). The standard deviation of measurements (SD) made in a given capillary, during single or multiple recording sessions were modest for Po_2_InterRBC (mean SD = 2.6 ± 0.3 mm Hg) and slightly larger for Po_2_RBC (mean SD = 5.4 ± 0.5 mm Hg). Figure 1C illustrates that average Po_2_ values masked the large span of all values measured. This was particularly true for Po_2_RBC, for which values frequently exceeded 70 mm Hg. Overall, these data show that in the glomerular layer of the awake resting mouse, interstitial Po_2_ ranges from 15 to 35 mm Hg in about 82% of our measurements. We then investigated whether the range and fluctuations of Po_2_values depend on two vascular parameters, RBC blood flow and hematocrit, in the same capillaries.

### Capillary blood flow and hematocrit in the olfactory bulb of the awake resting mouse

**Figure 2.**
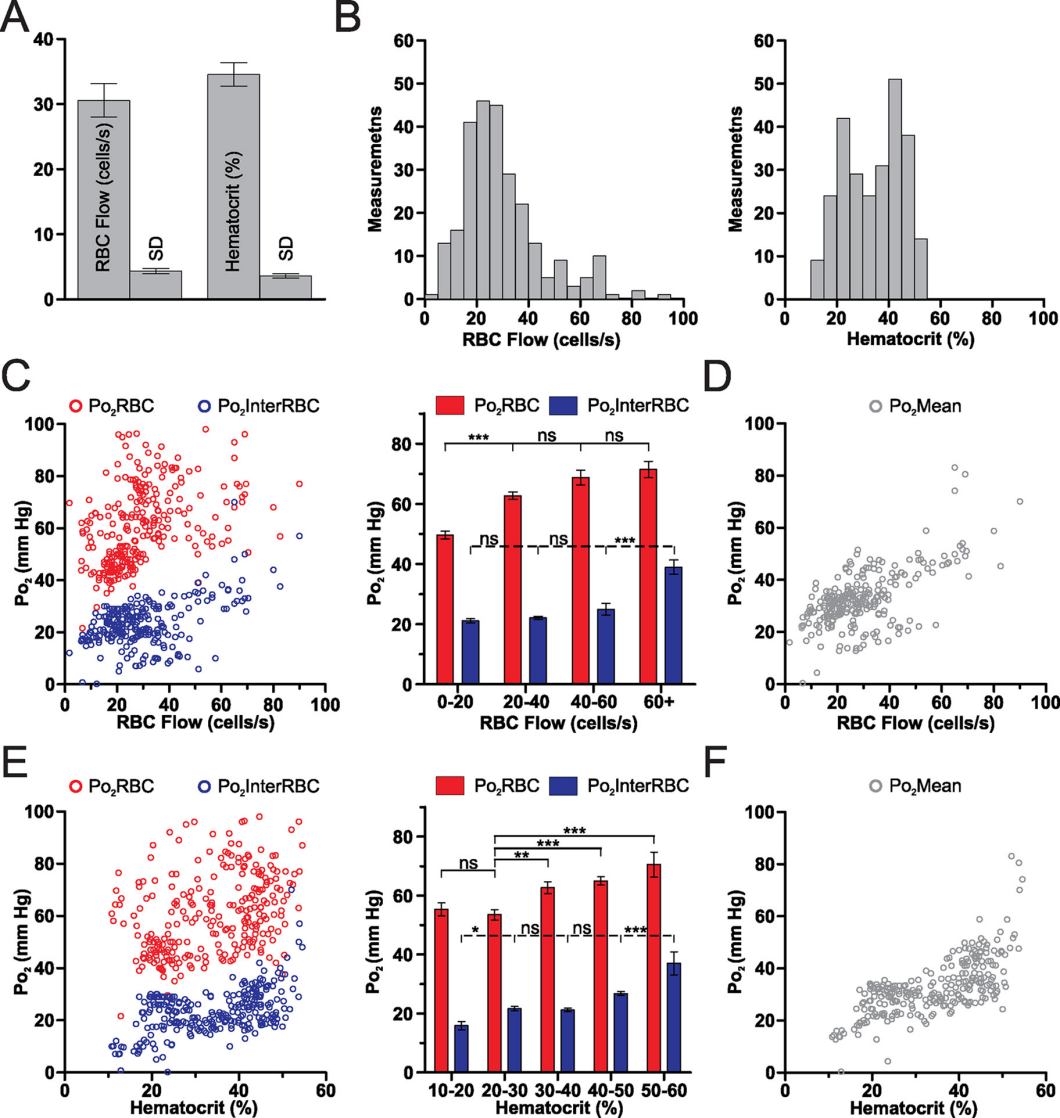
Relationships of capillary blood flow and hematocrit to Po_2_ values, in the olfactory bulb glomerular layer of the awake mouse. (**A**) RBC flow and hematocrit calculated from the mean values of each capillary (n = 38). SD (the average of the SD values for each capillary, presented as mean standard deviation ± SEM) illustrates the fluctuations of RBC flow and hematocrit in each capillary. (**B**) Frequency distributions of RBC flow and hematocrit (5% bin). (**C**) Distribution of all Po_2_RBC and Po_2_InterRBC measurements as a function of RBC flow. Note that the Po_2_InterRBC is independent of RBC flow below 60 cells/s while Po_2_RBC increases with RBC flow below 40 cells/s. (**D**) Po_2_Mean as a function of RBC flow. (**E**) Distribution of Po_2_RBC and Po_2_InterRBC as a function of hematocrit. Po_2_InterRBC is independent of hematocrit from 20 to 50%. Po_2_RBC increases with hematocrit at low values. (**F**) Po_2_Mean as a function of hematocrit. Bar graph data presented as mean ± SEM. *p<0.05, **p<0.01, ***p<0.001. Kruskal-Wallis test with 2-tailed Dunn's multiple comparison post-hoc test. For all plots n = 5 mice. **DOI:** http://dx.doi.org/10.7554/eLife.12024.004

Mean capillary RBC flow and hematocrit values were 30.6 ± 2.6 cells/s and 34.6 ± 1.8%, respectively (Figure 2A). Both RBC flow and hematocrit displayed a wide range of values (Figure 2B) with a positively skewed frequency distribution of RBC flow. Simultaneous measurements of Po_2_ and blood flow parameters revealed that although interstitial Po_2_ (Po_2_InterRBC) is correlated with both RBC flow and hematocrit (r = 0.3091, p<0.0001 and r = 0.4365, p<0.0001, Spearman r correlation, respectively), these relationships are non-linear. In particular, Po_2_InterRBC is mostly independent of both RBC flow below 60 cells/s and hematocrit from 20 to 50% (Figure 2C and E), increasing only at high values of both parameters. This stable region covers 82.4% of our measurements. In contrast, Po_2_RBC increased with RBC flow and hematocrit at low values, becoming stable above 20 cells/s and 30%, respectively (Figure 2C and E). Note that Po_2_Mean increased with both RBC flow and hematocrit values (RBC flow: r = 0.5116, p<0.0001; hematocrit: 0.6752, p<0.0001, Spearman r correlation, Figure 2D and F).

### Brain oxygenation is greatly enhanced by isoflurane anesthesia

**Figure 3.**
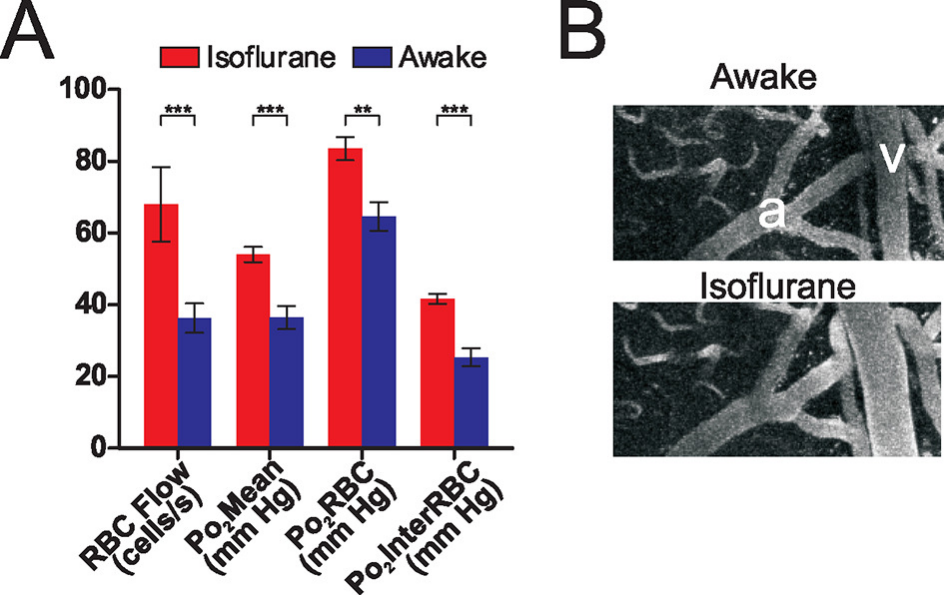
Isoflurane changes the brain oxygenation state. Po_2_ and RBC flow were compared in the same sets of olfactory bulb glomerular layer capillaries when the animal was awake, and when the animal was anesthetized with isoflurane (0.75%, delivered in air, no oxygen added). (**A**) Isoflurane anesthesia increased all RBC flow and Po_2_ values as compared to the awake state (n = 142 measurements in each condition, 16 capillaries from 3 mice). Bar graph data presented as mean ± SEM., ***p<0.001, paired 2-tailed Wilcoxon signed rank test. (**B**) Isoflurane anesthesia induced a large dilation of pial vessels (a: artery, v: vein). See also Figure 3—figure supplement 1. **DOI:** http://dx.doi.org/10.7554/eLife.12024.005

Isoflurane is a volatile anesthetic that is commonly used in the study of brain activation and metabolism. It differently affects regional cerebral blood flow in humans (Ramani and Wardhan, 2008), and modulates neurovascular coupling in a concentration-dependent fashion (Masamoto et al., 2009) as well as the relationship between spontaneous or evoked neuronal activity with BOLD signal (Aksenov et al., 2015). However, its effects on brain oxygenation have only been investigated using approaches with low spatial resolution and which did not allow simultaneous measurement of blood flow (Liu et al., 1995; Ortiz-Prado et al., 2010). We performed paired measurements of EATs and flow parameters in a set of capillaries, both when the animals were awake and when they were anesthetized with isoflurane (~0.75% as measured at the animal’s snout, delivered in air with no supplementary O_2_). As can be seen from Figure 3A, isoflurane significantly increases all capillary Po_2_ values (Po_2_Mean, Po_2_RBC and Po_2_InterRBC). This effect was present in all but one of the capillaries tested. Although reduced neuronal activity (and hence O_2_ consumption) in the isoflurane anesthetized state (Aksenov et al., 2015) is likely to play a role, it appears that this elevation of Po_2_ largely resulted from an increase in RBC flow, as the increase in Po_2_ values observed is in accordance with that which would be predicted from the observed increase in RBC flow based on the relationship presented in Figure 2C and D. This increase in capillary RBC flow rate is related to isoflurane’s vasodilatory effects on large vessels (Figure 3B) (Koenig et al., 1994). Consequently, cellular processes, including neuronal activity in response to odor, occur at a much higher oxygen concentration during isoflurane anesthesia than in the awake, resting state, and this difference is likely to be exacerbated when isoflurane is delivered in gas mixtures where [O_2_] is greater than 21% (as in many other studies). Since the olfactory bulb glomerular layer has a specific neuronal and vascular organisation that could be a main determinant of Po_2_ values, we extended our investigation of tissue oxygenation to the cerebral cortex, due to its importance to higher cognitive functions and its use as an ischemic model.

**Figure 3—figure supplement 1.**
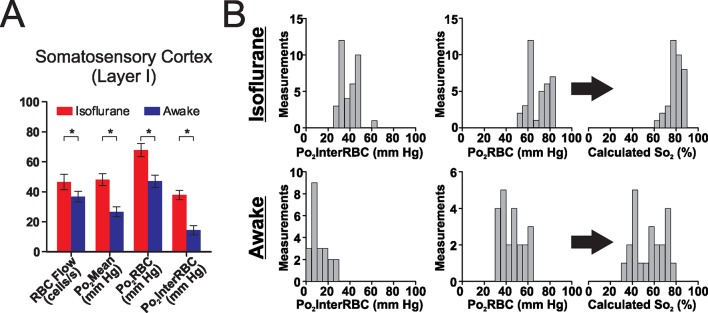
Isoflurane alters oxygenation in the somatosensory cortex. (**A**) As in the glomerular layer, isoflurane anesthesia (0.75% delivered in 21% O_2_ air) increases Po_2_ and RBC flow in layer I capillaries in the somatosensory cortex relative to the awake state. (**B**) This isoflurane-induced increase in Po_2_ was associated in a right-shift in the frequency distribution of measured Po_2_Mean, Po_2_InterRBC and Po_2_RBC along with that of estimated So_2_. *p<0.05, paired, 2-tailed, Wilcoxon signed rank test. n = 36 measurements under isoflurane and 22 in the awake state, from five capillaries. **DOI:** http://dx.doi.org/10.7554/eLife.12024.006

### Capillary flow and Po_2_ values in the somatosensory cortex of the awake resting mouse

**Figure 4.**
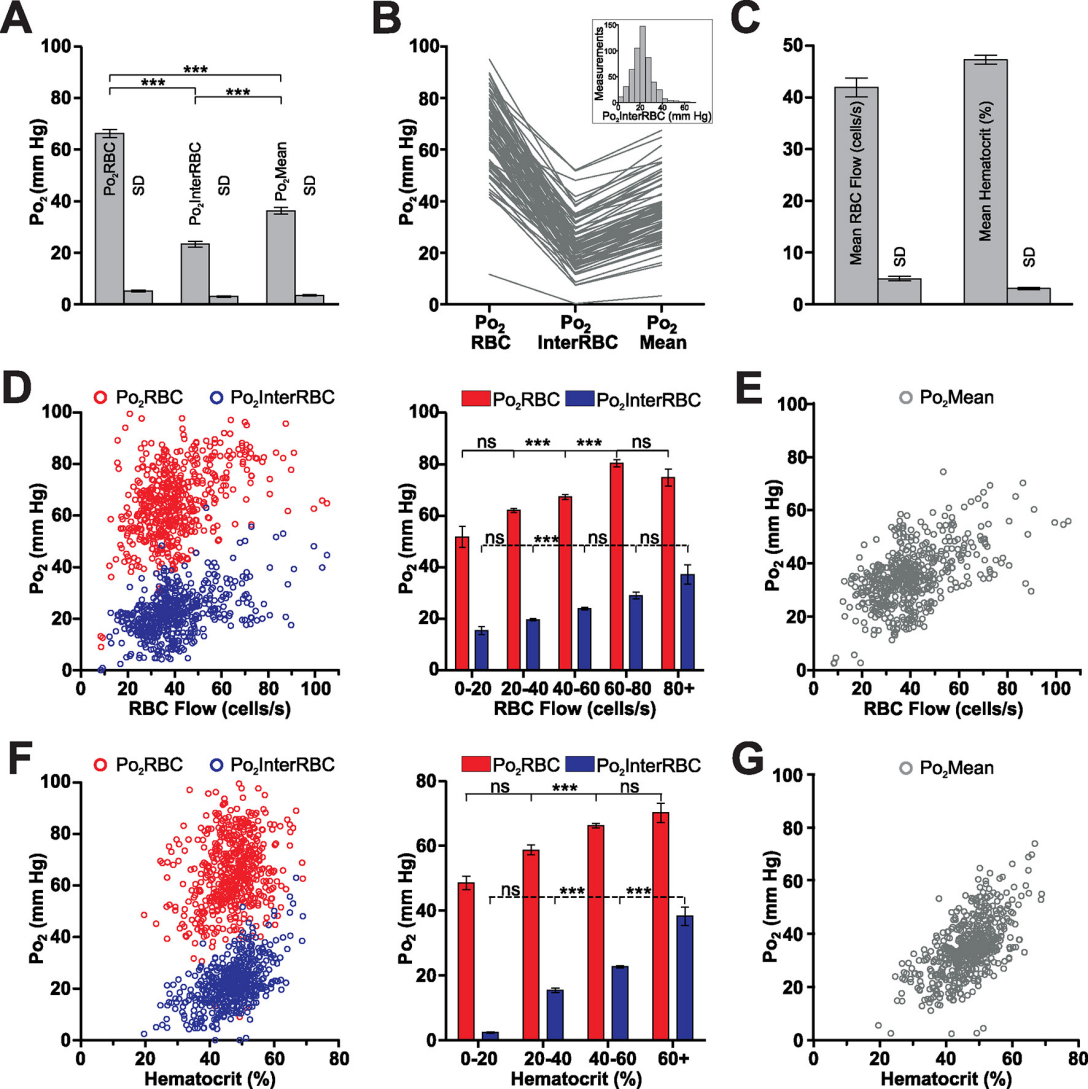
The relationship of Po_2_ to RBC flow and hematocrit in the somatosensory cortex of the awake mouse. (**A**) Average values and SD of Po_2_ parameters in somatosensory cortex capillaries, calculated from the mean values from each capillary (81 capillaries, 528 measurements, SD = the average of the SD values for each capillary, presented as mean standard deviation ± SEM). (**B**) Distribution of all capillary Po_2_ values averaged in (**A**). Frequency distribution histogram of local tissue Po_2_ (Po_2_InterRBC) in inset. 5 mm Hg bin. (**C**) Average values and SD of RBC flow and hematocrit calculated from the mean values from each capillary. (**D**) Distribution of all Po_2_RBC and Po_2_InterRBC measurements as a function of RBC flow. Note that for most values (from 20 to 60 cells/s), both Po_2_InterRBC and Po_2_RBC increase with RBC flow. (**E**) Po_2_Mean as a function of RBC flow. (**F**) Distribution of all Po_2_RBC and Po_2_InterRBC measurements as a function of hematocrit. For most values (from 20 to 60% ), both Po_2_InterRBC and Po_2_RBC increase with hematocrit. (**G**) Po_2_Mean as a function of hematocrit. Bar graph data presented as Mean ± SEM. *p<0.05, **p<0.01, ***p<0.001. Kruskal-Wallis test with 2-tailed Dunn's multiple comparison post-hoc test. **DOI:** http://dx.doi.org/10.7554/eLife.12024.007

We made measurements of all Po_2_ and blood flow parameters in capillaries distributed from the cortical surface to a depth of 410 µm in the fore- and hind-limb regions of the somatosensory cortex. Due to the greater inter-capillary distance in the cortex than in the glomeruli (Lecoq et al., 2009; Sakadžić et al., 2014), and as Po_2_ gradients have previously been observed around blood vessels in anesthetized animals (Devor et al., 2011; Sakadzić et al., 2010; Sharan et al., 2008), it is likely that there are tissue regions, in areas far from the nearest capillary, in which the Po_2_ is lower than what is reported by Po_2_InterRBC. Nonetheless, based on our previous results (Parpaleix et al., 2013) in olfactory bulb glomeruli and on theoretical predictions (Lücker et al., 2014), we expect that Po_2_InterRBC reports steady-state tissue Po_2_ up to a radius of approximately 10 µm from a capillary. Furthermore, recent work, that models hematocrit distribution in large microcirculatory networks and accurately replicates physiological RBC distribution, predicts that the distribution of oxygen in tissue volumes supplied by these networks is largely homogeneous (Gould and Linninger, 2015). We thus consider that our measured values of Po_2_InterRBC represent a significant proportion of the range of Po_2_ values present in the cortical interstitium. Our Po_2_ measurements revealed that the averages and ranges of Po_2_ values in cortical capillaries (Figure 4A and B) were similar to those measured in the olfactory bulb glomerular layer (Po_2_RBC = 66.3 ± 1.6 mm Hg, Po_2_InterRBC = 23.3 ± 1.1 mm Hg, and Po_2_Mean = 36.3 ± 1.3 mm Hg), indicating that at rest in the awake state, these two brain areas have similar levels of both RBC, capillary and pericapillary oxygenation. In contrast, the average RBC flow and hematocrit values (41.9 ± 1.8 cells/s and 47.3 ± 0.9%, respectively) were significantly higher than those found in the olfactory bulb glomerular layer (Figure 4B) with the hematocrit values being higher than previously reported levels in the cerebral cortex of anesthetized animals (Hudetz, 1997). The lower hematocrit levels in glomerular layer capillaries could be related to differences in the bulb cerebrovascular supply which, in contrast to the cortex (Blinder et al., 2013), is poorly established (Coyle, 1975).

As in the olfactory bulb, both Po_2_RBC and Po_2_InterRBC were correlated with RBC flow rate and hematocrit (Po_2_RBC with RBC Flow and hematocrit: r = 0.3824, p<0.0001 and r = 0.2315, p<0.0001, Spearman r correlation, respectively; Po_2_InterRBC with RBC Flow and hematocrit: r = 0.4283, p<0.0001 and r = 0.5018, p<0.0001, Spearman r correlation, respectively). Po_2_InterRBC (Figure 4D and F) increased with RBC flow, from 20 to 60 cells/s, that is, over the majority of the measurements, becoming stable thereafter. It also increased with hematocrit. Similarly, Po_2_RBC increased with both RBC flow and hematocrit. Note that Po_2_Mean, was correlated with and increased through the entire range with both RBC flow and hematocrit (RBC flow: r = 0.4670, p<0.0001; hematocrit: 0.5945, p<0.0001, Spearman r correlation, Figure 4E and G).

### Laminar organisation of capillary blood flow and Po_2_ values in the superficial layers of the somatosensory cortex

**Figure 5.**
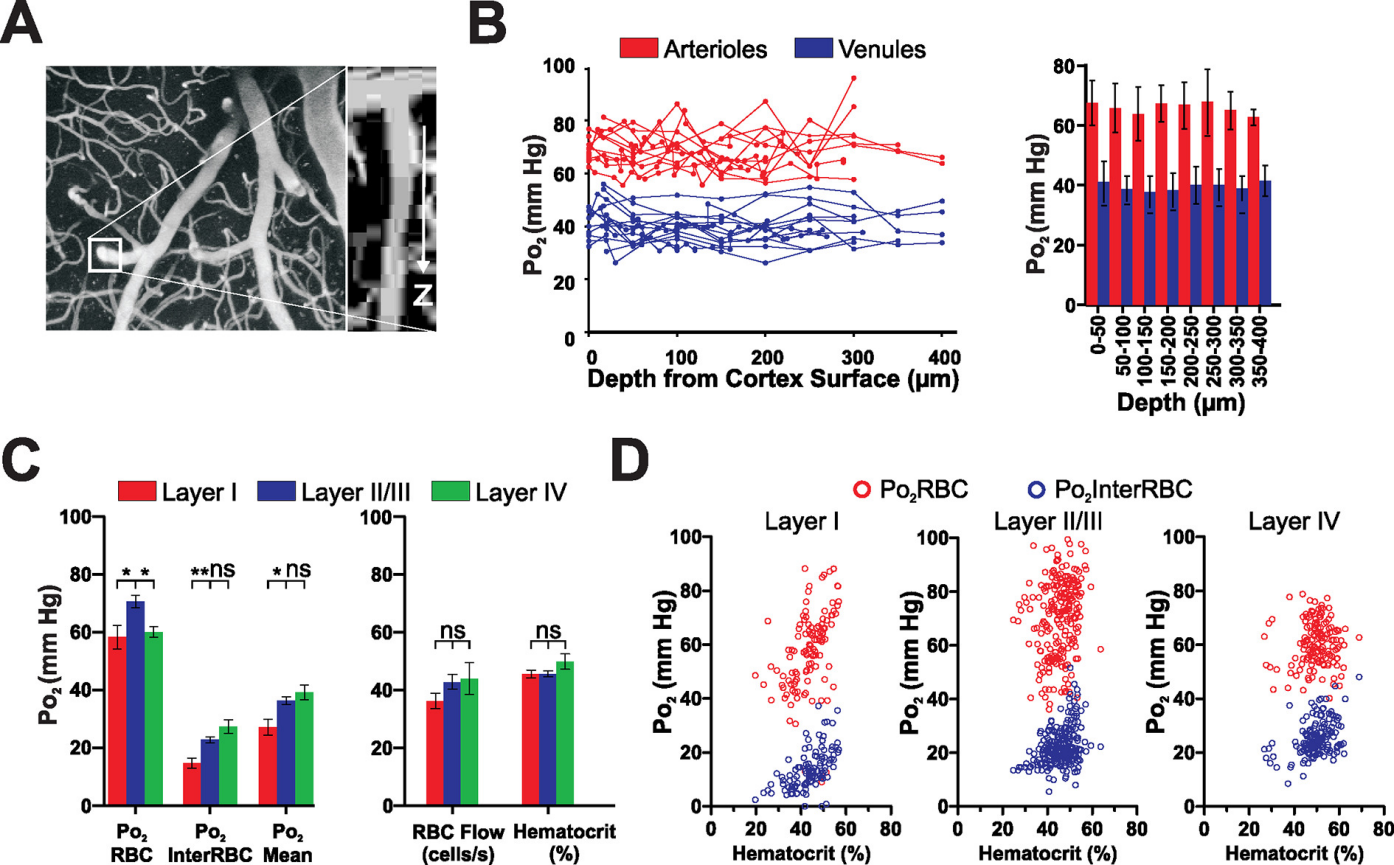
Depth profiles of vascular and local tissue oxygenation in the somatosensory cortex of the awake mouse. (**A**) Left panel, maximum intensity projection of superficial portion of the vasculature of the somatosensory cortex, with boxed area highlighting a penetrating arteriole shown in the XZ projection (right panel). (**B**) Left panel, Po_2_ values in penetrating arterioles (red) and venules (blue) as a function of depth from the cortical surface. Each line represents a single vessel. Right panel, mean of all Po_2_ values recorded from vessels as a function of depth. Note the absence of Po_2_ gradients with depth (50 µm bin size, 136 measurements in 11 arterioles, 148 measurements in 14 venules, from 6 mice.) Data presented as mean ± SD (**C**) Comparison of Po_2_ values (left panel), RBC flow and hematocrit (right panel) in layers I, II/III and IV. Note that capillary Po_2_Mean and Po_2_InterRBC are higher in layers II/III and IV than in layer I, although there are no significant differences in either blood flow parameter. Data presented as mean ± SEM. * p<0.05, **p<0.01, Kruskal-Wallis test with 2-tailed Dunn's multiple comparison post-hoc test. n = Layer I: 113 measurements in 17 capillaries, Layer II/III: 230 measurements in 41 capillaries, Layer IV: 151 measurements in 15 capillaries (**D**) Po_2_RBC and Po_2_InterRBC as a function of hematocrit in layers I, II/III and IV. **DOI:** http://dx.doi.org/10.7554/eLife.12024.008

Several studies, using polarographic electrodes or 2PLM in anesthetized rodents, have reported that vascular and interstitial Po_2_ varies with cortical depth (Devor et al., 2011; Masamoto et al., 2003; Sakadzić et al., 2010). As anesthetics could differently modulate synaptic activity and blood flow in layers I to IV, it is difficult to predict the extent to which Po_2_ depth variations occur in the awake animal. We thus first measured Po_2_ in descending and ascending large vessels and then compared Po_2_and blood flow parameters of capillaries from layers I to IV (from the surface to 410 µm in depth). Capillaries less than 60 μm below the surface were considered to be in layer I, those from 90 to 260 μm below the surface were classified as layer II/III capillaries and those deeper than 340 μm were considered layer IV capillaries (capillaries from 260 to 340 µm were not considered due the ambiguity of their location). Penetrating vessels (arterioles and venules) were traced from their point of descent below the surface down along their main trunk until they ramified into smaller vessels (or descended below 410 µm, which was the maximum depth at which we made measurements). Note that in these vessels EATs were not detectable due to the close apposition of RBCs. The mean Po_2_ was 69.2 ± 1.4 mm Hg for arterioles and 39.9 ± 1.3 mm Hg for venules and, in contrast to what was reported in anesthetized animals (Devor et al., 2011; Sakadzić et al., 2010), no gradient was observed with depth (Figure 5A and B).

Laminar analysis of capillary blood flow and Po_2_ values revealed some specific differences: although the hematocrit and blood flow values were similar in all three layers (Figure 5C, right panel), all Po_2_ values were lower in layer I than in layer II/III (Figure 5C, left panel). The low Po_2_InterRBC values 14.7 ± 1.7 mm Hg suggests that interstitial Po_2_ is correlated with the capillary density which is lower in layer I than II/III (Blinder et al., 2013; Sakadžić et al., 2014). The correlated analysis of Po_2_ with hematocrit revealed that low Po_2_InterRBC values were present at a wide distribution of hematocrit levels (Figure 5D, left panel). In addition, even though the average Po_2_RBC was higher in layer II/III than in layer IV, it was independent of hematocrit in both layers (Figure 5D). Finally, the effects of isoflurane in the cortex (Layer I, Figure 3—figure supplement 1) were similar to those observed in the olfactory bulb. Thus, in addition to its direct effects on neurons, isoflurane increases oxygen delivery to the entire brain.

### Regions of low tissue Po_2_ are present at rest

**Figure 6.**
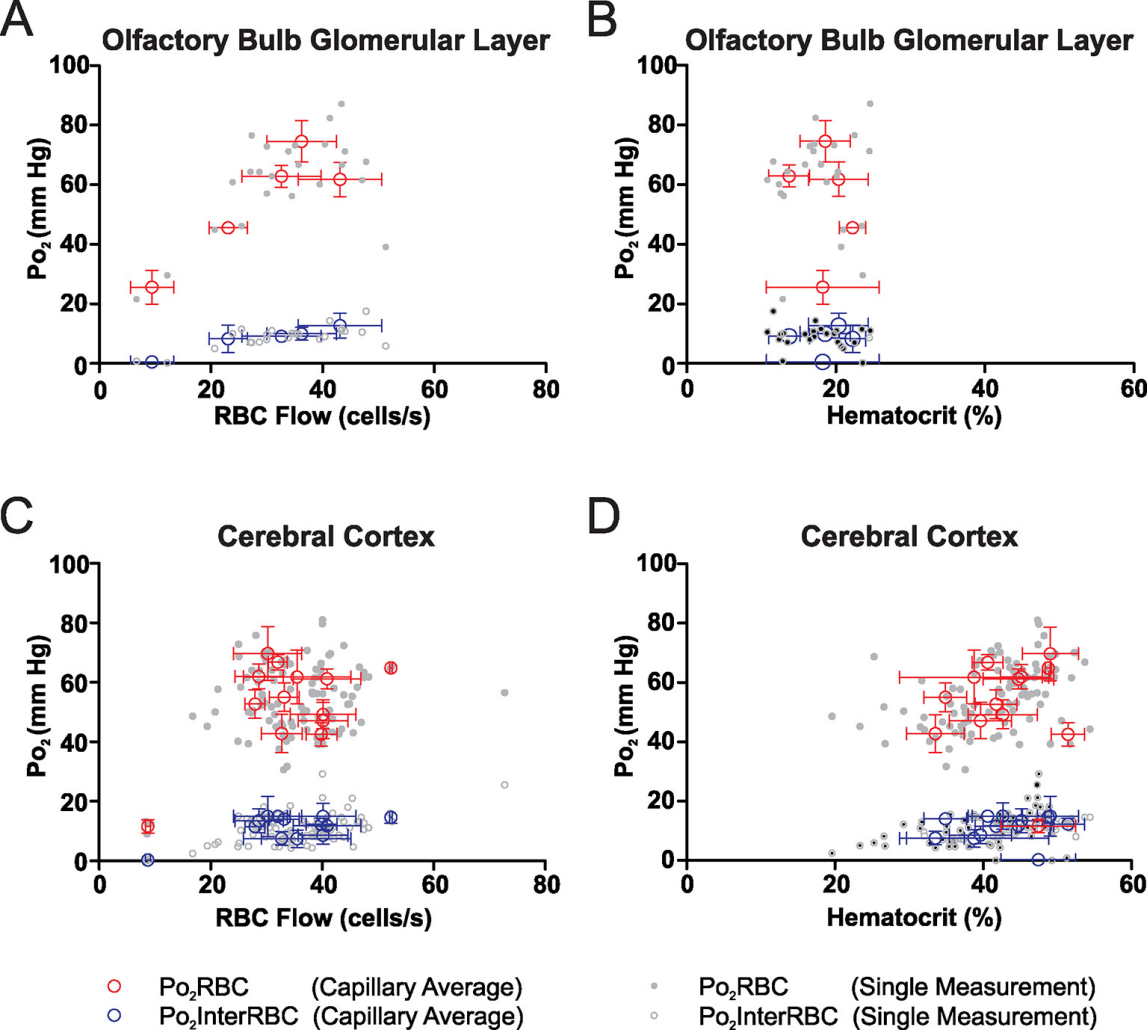
In the awake mouse, low interstitial Po_2_ in the olfactory bulb glomerular layer is associated with low hematocrit capillaries. Po_2_ values from all glomerular layer capillaries with average Po_2_InterRBC values of <15 mm Hg (n = 24 measurements in capillaries) are plotted as a function of RBC flow (**A**) and hematocrit (**B**). The RBC flow rates in these capillaries were distributed across a wide range, whereas in all cases capillary average hematocrit was <25%, suggesting that, in the glomerular layer, areas of low interstitial Po_2_ are supplied by capillaries with relatively low hematocrit values (hematocrit of these capillaries = 18.6 ± 1.4%, n = 5; hematocrit of all other capillaries = 37 ± 1.7%, n = 33; p = 0.002, unpaired t-test. mean ± SEM). Conversely, cortical capillaries with average Po_2_InterRBC <15 mm Hg (n = 13 capillaries, 101 measurements) had wide ranges of both RBC flow (**C**) and hematocrit (**D**). However, the majority (10 of 13) of these capillaries were located in layer I. In all plots, single measurement values and mean ± SD of all measurements in each capillary shown. **DOI:** http://dx.doi.org/10.7554/eLife.12024.009

Po_2_InterRBC values show a wide distribution in both the olfactory bulb glomerular layer and the somatosensory cortex (Figure 1C and 4B). Notable in both structures is the presence of measurements of Po_2_InterRBC, and hence local tissue Po_2_, that were <15 mm Hg. In the olfactory bulb glomerular layer these low Po_2_InterRBC capillaries (n=5) were found to have a large range of RBC flow rates but low hematocrit levels (<25%, Figure 6A and 6B). In contrast, in somatosensory cortex capillaries (n = 13) in which the Po_2_InterRBC value was <15 mm Hg, neither RBC flow rates nor hematocrit levels were notably low (Figure 6C and 6D). Instead, these low Po_2_InterRBC values were mostly found in capillaries in layer I (10 of 13 capillaries), suggesting that the presence of low tissue Po_2_ values results from several factors.

### RBC hemoglobin is largely saturated in cerebral capillaries

**Figure 7.**
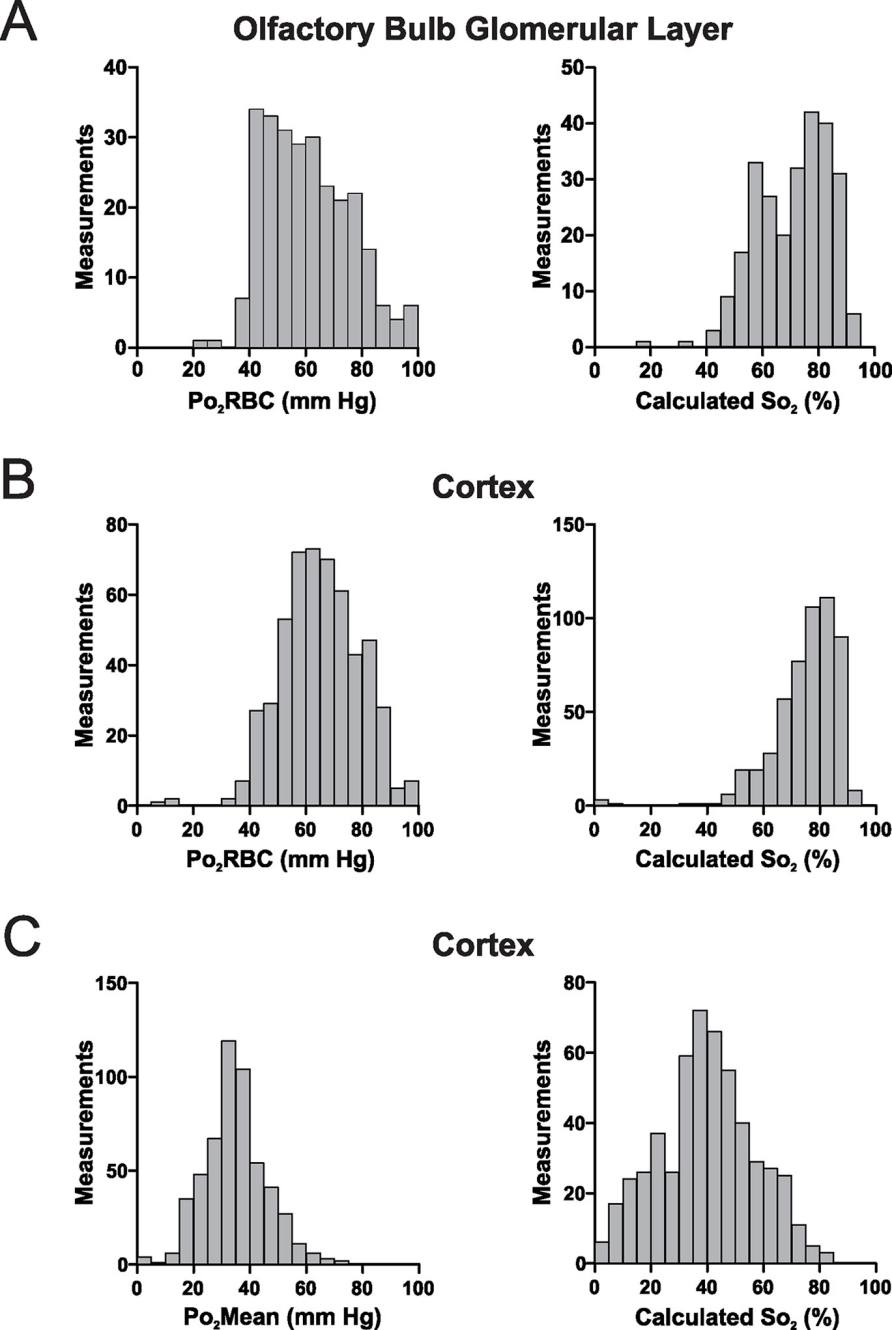
In the awake mouse, the majority of hemoglobin in cerebral capillaries is oxygenated. (**A**) Left panel, frequency distribution of Po_2_RBC values measured in the glomerular layer of awake mice, which were used to compute So_2_ values (right panel). (**B**) Equivalent Po_2_RBC (left panel) and So_2_ distributions (right panel) from the cerebral cortex. In both structures > 90% of the measurements have So_2_ values of >50% (94.7% and 98.5% of measurements in the glomerular layer and the cortex respectively) with ~60% of So_2_ values in the cortex being >75%. (**C**) Left panel, frequency distribution of Po_2_Mean values from cerebral cortex capillaries, with the So_2_ distribution that would be computed were these lower Po_2_ values considered to represent those present at the hemoglobin molecules in RBCs (right panel). Glomerular layer: n = 262 measurements in 38 capillaries. Somatosensory cortex: n = 528 measurements from 81 capillaries. Bin size 5 mm Hg and 5% for Po_2_ and So_2_ respectively. **DOI:** http://dx.doi.org/10.7554/eLife.12024.010

Given that Po_2_RBC should be representative of the Po_2_ level inside RBCs, we used known values of the Hill coefficient and P_50_ for mouse hemoglobin (Uchida et al., 1998) to estimate hemoglobin saturation (So_2_) in capillaries. In both the olfactory bulb glomerular layer and the somatosensory cortex (Figure 7A and B), measured So_2_ is >50% in the vast majority of cases (94.7% and 98.5% of measurements in the glomerular layer and the cortex, respectively). This shows that in the resting brain, the majority of hemoglobin exists as oxyhemoglobin. Furthermore the presence of numerous measurements of So_2_ >85% in both structures suggests that hemoglobin saturation is nearly maximal in a significant proportion of capillaries (~10% of capillaries in the glomerular layer, ~20% in the somatosensory cortex). Note that, if So_2_ were to be inappropriately estimated from Po_2_Mean values, a very different distribution of hemoglobin saturation would be derived (Figure 7C). This demonstrates that in capillaries, an accurate measurement of Po_2_RBC is a prerequisite for estimating So_2_.

## Discussion

In the olfactory bulb and somatosensory cortex of awake resting mice, we report similar average values of interstitial Po_2_ (~23 mm Hg). In the awake rodent, brain oxygenation has previously only been investigated with lower resolution approaches. Liu et al. (Liu et al., 1995) used electron paramagnetic resonance (EPR) oximetry with a lithium phthalocyanine crystal being implanted in the cerebral cortex of rats 24 hr prior to the measurements. They reported that restrained and untrained rats have a cerebral Po_2_ of about 34 mm Hg, which, surprisingly, was reduced by isoflurane anesthesia (1% in 21% O_2_) to about 24 mm Hg. This resting value (34 mm Hg) is significantly higher than our average interstitial Po_2_ value, most probably due to the effects of the stress resulting from restraint in the absence of habituation. The effect of isoflurane, which decreased Po_2_ is intriguing, and indicates that the main effect of isoflurane in this case was to abolish the Po_2_ augmenting effects of stress. Interestingly, increasing the delay from crystal implantation to 7 days and introducing some habituation to restraint reduced cerebral Po_2_ to 27 mm Hg (Dunn et al., 2000). The best demonstration of physiological measurements of Po_2_ has been performed in unrestrained rats, in which brain Po_2_was measured with an implanted fiber optic probe measuring quenching of an oxygen sensor (Ortiz-Prado et al., 2010). Although the probe was very invasive, it reported an average bulk tissue Po_2_ value of 25 mm Hg, a value very similar to ours. We are thus confident that Po_2_InterRBC is an excellent reporter of the interstitial Po_2_, that our extensive habituation procedures are efficient, and that this study gives the first non-invasive and physiological values of Po_2_, hematocrit and capillary blood flow in the rodent brain.

Our results show a number of differences from those previously reported with high-resolution measurement techniques in anesthetized animals. Tissue Po_2_ values have previously been reported to be in the range of ~5–100 mm Hg, with the higher values occurring in regions close to pial arterioles (Devor et al., 2011; Sakadzić et al., 2010). Our values of tissue Po_2_ (Po_2_InterRBC) lie in the lower end of this wide range, do not include measurements from the capillary-free peri-arteriolar regions, and so are likely to be reflective of the Po_2_ levels that exist in the majority of the tissue, away from large arterioles. Considering intravascular Po_2_, our measurements of capillary Po_2_Mean (average ≈36 mm Hg in the cortex) seem to be broadly in agreement with those reported by Sakadzić et al., 2010 (~25–35 mm Hg), but our recorded values of capillary RBC Po_2_ (Po_2_RBC, average ≈66 mm Hg in the cortex), are dramatically higher than those published by Sakadzić et al., 2014, where the most frequent values were ~25–35 mm Hg. A prominent discrepancy between the results of previous studies and our findings in awake, resting animals relates to changes in cortical oxygenation with depth. Many previous studies report drops in interstitial Po_2_ with increasing depth in the cortex (Cross and Silver, 1962; Devor et al., 2011; Masamoto et al., 2003; Nair et al., 1975; Ndubuizu and LaManna, 2007; Whalen et al., 1970), with Po_2_ in layer I being higher than in underlying layers. In contrast, our measures of Po_2_InterRBC suggest that interstitial Po_2_ is lower in layer I than in either layer II/III or IV. These conflicting patterns of laminar variations of Po_2_ also exist in comparisons of the mean vascular Po_2_ values (Po_2_Mean), where previous 2PLM studies have reported decreases in mean vascular Po_2_ in concert with decreases in penetrating arteriole and venule Po_2_ (Devor et al., 2011; Sakadzić et al., 2010) with increasing depth in the cortex. In the present study, we see an increase in capillary Po_2_Mean from layer I to deeper layers, and no gradient in penetrating vessel Po_2_ with depth. It is possible that the deviation from normal physiological conditions inherent in anesthesia and acute surgical preparation, which we avoid with our approach, leads to the emergence of these disagreements.

Although average Po_2_InterRBC values in both the cortex and glomerular layer were ~23 mm Hg, in both structures a number of capillaries were found to have Po_2_InterRBC values <15 mm Hg, indicating the existence of regions in both structures where the interstitial Po_2_ is close to reported values of Po_2_ below which cellular respiratory rate is strongly dependent on Po_2_ (~10 mm Hg) (Kasischke et al., 2011). Furthermore, examples were found in both brain regions where the Po_2_InterRBC was below the value of ~3.4 mm Hg that has previously been reported as the critical Po_2_ in brain tissue (Kasischke et al., 2011). Similarly low tissue Po_2_ measurements have previously been observed (Ndubuizu and LaManna, 2007; Whalen et al., 1973), but their existence has been interpreted as being related to disruption of normal tissue physiology in the experimental preparations (Wilson et al., 2012). However, the presence of such low values in our awake preparations indicates that, surprisingly, at least some small regions of the brain can subsist at very low Po_2_ values.

Our finding of high Po_2_RBC and corresponding capillary So_2_ (estimated using the parameters used in Sakadžić et al., 2014) values in both the olfactory bulb and the somatosensory cortex (Figure 7) differs from recently published findings in the anesthetized, ventilated mouse (Sakadžić et al., 2014). We find that capillary So_2_ in both structures is generally high, indicating that the majority of hemoglobin in these vessels exists as oxyhemoglobin, and thus that capillaries are capable of supplying very significant quantities of oxygen to support neural function.

In conclusion, the present study establishes, for the first time, accurate and precise values of physiological Po_2_ in the vasculature and interstitium of mouse cerebral grey matter. As it is known that O_2_ concentration is a critical parameter in determining the properties of neuronal function (Huchzermeyer et al., 2008, 2013; Ivanov and Zilberter, 2011), and neurovascular interactions (Gordon et al., 2008), these values provide a standard on which future research can rely to provide relevant, physiologically accurate conditions of oxygenation in which to investigate such processes.

## Materials and methods

### Experimental procedures

#### Animal preparation and surgery

All animal care and experimentation was performed in accordance with the INSERM Animal Care and Use Committee guidelines. Adult C57BL/6 mice (3–6 months old, 25-–35 g, both males and female, housed in 12-hr light-dark cycle) were used in this study (n = 5 mice for olfactory bulb capillaries, n = 3 mice for cortex capillaries). Chronic glass cranial windows were implanted over the area of interest, either the olfactory bulb or the somatosensory cortex, with great care taken to not disturb the dura mater. The animals received anti-inflammatory and analgesic treatment (Carprofen, one daily 0.15 mg subcutaneous injection, administered pre-surgically and for the three days post-surgery), antibiotics (Ceftrioxone, one daily 0.25 mg subcutaneous injection, administered pre-surgically and for the three days post-surgery) and dexamethasone to reduce cerebral edema (one daily 60 µg subcutaneous injection on the day before surgery, directly before the surgery and the first post-surgical day). Surgical anesthesia was induced with ketamine-xylazine (100 mg and 8 mg per kg body mass, respectively). During surgery, the mice breathed a mixture of air and supplementary oxygen (the final inhaled proportion of oxygen was ~30%) and the body temperature monitored by a rectal probe and maintained at ~36.5°C by a feedback-controlled heating pad. A craniotomy was performed with great care taken not to apply great pressure to the bone and the area was regularly flushed with cool aqueous buffer solution to avoid damage or heating of the underlying tissue. Cover glass (~150 µm thick) was used for the window and sealed in place with photopolymerizable dental cement (Tetric EvoFlow, Ivoclar Vivadent, Schaan, Principality of Liechtenstein), which was also used to form a head-cap in which a titanium head-bar was also embedded.

#### Habituation of mice to head restraint

In the weeks preceding surgery, the animals were supplied with a treadmill (Fast-Trac, Bio-Serv, Flemington, New Jersey) in their cages that was similar to that which forms part of the restraint apparatus used during 2PLM recording (see below). In the days that preceded the surgery, the mice were gently habituated to handling, and provided with treats (sugar pellets, Bio-Serv). 2–3 days after the surgery restraint habituation began. The frame used for head restraint during the habituation and imaging consists of a metal frame in which the mouse’s head-bar is secured, and a treadmill wheel (similar to that in the animal’s home cage). This configuration allows the animal limbs and body to move freely while the head is stably fixed. All restraint-habituation sessions were carried out in the set-up that was used for imaging, with the animal kept in the near-dark at all times. Habituation sessions were performed multiple times per day over the course of 2–4 weeks, with the duration increasing from 5 min to >1 hr. Animals were considered ready for use in experiments when they could be easily fixed in the recording apparatus while awake, and their behavior during the sessions consisted of short bouts of locomotion (~30 s) separated by longer periods of stillness (5–15 min) during which measurements were performed.

On the days on which recordings were taken, the animals were briefly anesthetized with isoflurane (2%, in air. Total duration of anesthesia <5 min), and PtP-C343 solution (Mw≈65 kDa, 2.5 mM in 0.9% saline) was introduced intravenously via retro-orbital injection. Fluorescein isothiocyanate dextran (Mw = 150 kDa) was often co-administered with PtP-C343 to enhance imaging contrast. The final PtP-C343 concentration in the plasma is estimated to be ~100 µM. After the injection, the mice were returned to their home cages for ~90 min, before being brought back to the experimental room, for ~30 min before the start of the recording session. We implemented this delay of ~2 hr between the injection and the following recording session to avoid potential confounding effects of the brief exposure to isoflurane or the effects of the injection itself. We found that the concentration of PtP-C343 in the blood typically remained sufficient for Po_2_ measurements to be made over 1–3 days after an injection, allowing for multiple recording sessions to be made before reinjection was necessary. Each recording session lasted from 1–3 hr, depending on the type of measurements performed and the behavior of the animal, and each animal underwent 1–3 recording sessions per day over the course of 2–7 days, with breaks of at least 1–3 hr between each session. The data derived from each of the recording sessions across the 1–2 days post-injection was similar, indicating that no lingering effects of the injection procedure or brief isoflurane anesthesia had affected the measurements in the first recording session post-injection.

#### 2PLM setup and procedure

The 2PLM setup is as described in Lecoq et al. (2011), and Parpaleix et al. (2013). Briefly, the output from a Ti:sapphire laser (Mira, Coherent, Santa Clara, California; λ = 850 nm, 120 fs pulse width, 76 mHz) is gated by an acousto-optic modulator (AOM, AA Optoelectronic, Orsay, France; MT110-B50-A1.5-IR-Hk). This allows for repetitive cycles of alternating 'on' and 'off' periods to be generated, that correspond to periods of excitation and recording from the PtP-C343 probe in the sample. The excitation period was 24 µs and the recording period was 225 µs, for a total cycle period of 250 µs and a repetition rate of 4 kHz. The scanning of the excitation light by the galvanometric mirrors (Cambridge Technology, Bedford, Massachusetts) is synchronized with the gating of the laser output by the AOM, using custom-built electronics and LabVIEW software (National Instruments, Austin, Texas). The excitation light was focused with a water-immersion objective lens (either x63 Leica [Wetzlar, Germany] or x40 Olympus lens [Olympus, Tokyo, Japan]).

Emitted photons were divided by a dichroic mirror (cut off wavelength = 560 nm). The green channel was bandpass filtered (HQ 520/40 m, Chroma Technology Corp, Bellows Falls, Vermont). The red channel had two shortpass filters (FF01-750/sp-25, Semrock, Rochester, New York), and one bandpass filter (HQ 680/60 m-2P, Chroma Technologies Corp), and a red-sensitive photomultiplier tube (PMT, R10699, Hamamatsu, Hamamatsu City, Japan). PMT signals were amplified with custom-build electronics and sampled at 1.25 MHz by an acquisition card. Photons detected in the green channel during the on phase of the recording cycle were used to detect the borders of RBCs and extract RBC flow rates and hematocrit values (see below). Phosphorescence decays detected during the 'off' period were averaged over a number of cycles (~5000–50,000 decays) and the lifetime of the phosphorescence determined by fitting a single exponential curve to the data. The first 6–7 bins (~5 µs) after the end of the on-phase were discarded. This lifetime measurement is then converted to a value of Po_2_ using a calibration curve. We used the nonlinear least-squares method and the Marquardt-Levenberg algorithm to obtain the decay lifetimes and associated standard errors. We used a Stern-Volmer–like calibration curve to convert the phosphorescence lifetimes into the corresponding Po_2_ values. This analysis was performed using custom build software (see below) developed using LabView (National Instruments).

#### Capillary blood flow analysis and EAT properties extraction

Initially, fast 3D image acquisition was performed to identify and target vessels of interest. Po_2_ measurements were then made by focusing the excitation point in blood vessels of interest, and recording the fluorescence and phosphorescence emitted from 10,000–40,000 cycles of excitation and detection of phosphorescence decays (2.5–10 s of measurement) at each point. Typically 4–8 measurements were performed in each capillary, with the total number of decays recorded for each capillary being around 60,000–100,000.

Details of the methods of determining EAT properties, RBC flow rates and hematocrit can be found in Parpaleix et al. (2013) and Lecoq et al. (2011), see especially Figures 1 and 3 therein). In brief, the fluorescence recorded during the excitation period of the cycle was analyzed and the passage of RBCs through the excitation point detected based on the associated dips in fluorescence intensity. These transient reductions in fluorescence intensity were detected and quantified using a binary threshold method, with the borders of the RBC corresponding to the full width at half maximum of the fluorescence dip. This allowed for the measurement of both capillary RBC flow rate (the number of RBCs detected per unit time) and hematocrit (the number of decay cycles originating from within RBCs as a percentage of the total number of decay cycles). The borders of the detected RBCs were used as time-stamps, and the phosphorescence decays recorded were binned according to their distance in time from the borders of the nearest RBC. The Po_2_RBC value was calculated from the average lifetime of decays recorded in the 1–4 ms around the border of the RBC, whereas the Po_2_InterRBC was determined from average value of decays at mid-distance between RBCs (averaged over a window of at least 5 ms). The EAT-extraction custom-built software is now available online at the following address: https://github.com/charpak-lab/EAT-detection. Hemoglobin saturation (So_2_) was estimated from Po_2_RBC using the Hill equation, with a Hill coefficient (2.59) and P_50_ (40.2 mm Hg), which are accurate for C57BL/6 mice (Uchida et al., 1998) and have previously been used to make estimations of mouse cerebral So_2_ from Po_2_ data (Sakadžić et al., 2014).
